# A self-powered and drug-free diabetic wound healing patch breaking hyperglycemia and low H_2_O_2_ limitations and precisely sterilizing driven by electricity†

**DOI:** 10.1039/d2sc04242h

**Published:** 2022-09-30

**Authors:** Linlin Wang, Qiwen Su, Yi Liu, Tajiguli Yimamumaimaiti, Dandan Hu, Jun-Jie Zhu, Jian-Rong Zhang

**Affiliations:** Shaanxi Key Laboratory of Chemical Additives for Industry, College of Chemistry and Chemical Engineering, Shaanxi University of Science and Technology Xi'an 710021 China; State Key Laboratory of Analytical Chemistry for Life Science, School of Chemistry and Chemical Engineering, Nanjing University Nanjing 210023 P. R. China jjzhu@nju.edu.cn jrzhang@nju.edu.cn

## Abstract

Accelerating diabetes-related chronic wound healing is a long-sought-after goal in diabetes management. However, therapeutic strategies based on antibiotics or catalysts still face great challenges to break the limitations of antimicrobial resistance, low H_2_O_2_ and the blocking effect of bacterial biofilms on antibiotic/catalyst penetration. Herein, we reported a glucose biofuel cell-powered and drug-free antibacterial patch, which consisted of an MAF-7 protected glucose oxidase/horseradish peroxidase anode and a horseradish peroxidase cathode, for treating diabetic wounds. This self-powered patch could take high blood glucose as fuel to generate electricity and abundant reactive oxygen species (ROS) *in situ*, synergistically regulating local hyperglycemia and breaking the limitations of insufficient ROS caused by low H_2_O_2_ levels. In particular, the electric field created by the GBFC could drive the negatively charged bacteria to adhere firmly to the electrode surface. As a result, the ROS produced *in situ* on the electrodes was localized to the bacteria, realizing precise sterilization. *In vivo* experiments confirmed that this self-powered patch enabled the wounds on diabetic mice to take a mere 10 days to eliminate inflammation and form mature skin with new hair follicles, demonstrating its great potential in treating bacteria-infected diabetic wounds.

## Abstract 2

A GBFC-powered antibacterial patch which can break low H_2_O_2_ limitations and precisely sterilize driven by electricity was created to treat bacteria-infected diabetic wounds without depending on any exogenous drugs.

## Graphical abstract

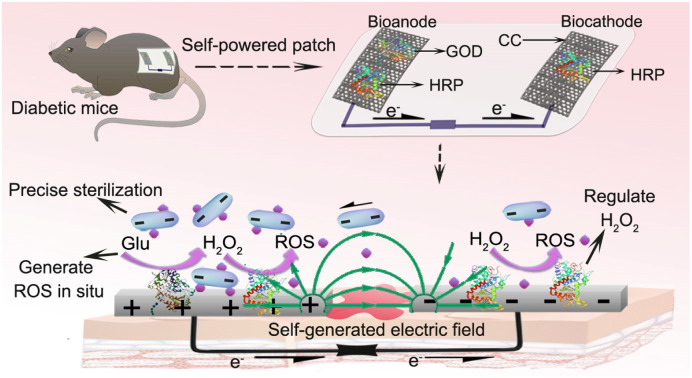

## Introduction

The rapid healing of diabetic wounds has become a worldwide problem.^1^ Compared to normal wounds, diabetic wound healing is much more difficult. Because hyperglycemia significantly retards the process of hemostasis and new blood vessel formation, it further prolongs the wound healing time.^2–4^ The prolonged exposure of the wound, together with hyperglycemia, leads to the spread of bacterial infection. Meanwhile, hyperglycemia causes the spillover of reactive oxygen (H_2_O_2_), which is a crucial contributor to the severely weak immune system of diabetes.^1,2,5^ Thereby, the exposed diabetic wounds undergo severe inflammation and spread bacterial infection, which will further lead to more severe wound ulceration or even amputation. Therefore, an effective antibacterial approach for diabetic wound treatment is urgently needed.

In this context, the antibacterial patches loaded with antibiotic drugs have been reported. However, besides the side effects on the viscera, antibiotics also induce the occurrence of drug resistance. What's more, the formation of bacterial biofilms induced by hyperglycemia severely prevents antibiotics from penetrating deep wounds.^6–8^ Given this, enzyme or enzyme-like catalysts, that can catalyze the decomposition of H_2_O_2_ to produce reactive oxygen species (ROS), have been widely used in designing antibacterial patches, as the ROS has proven to be able to achieve the goal of non-resistant sterilization.^9–12^ Nevertheless, catalyst-based antibacterial patches still face four formidable challenges in accelerating diabetic wound healing: (i) the limited local H_2_O_2_ source severely limits the antibacterial effect and durability;^1,13,14^ (ii) the diffusion distance of ROS is known to be extremely short, resulting in significantly reduced antibacterial activity;^9^ (iii) hyperglycemia is the root cause of bacterial infection spread and it is difficult for the reported antibacterial patches without a glucose-lowering function to fundamentally eliminate the risk of bacterial infection and spread;^2,13^ (iv) the surface proteins expressed by different species of bacteria are quite different, thereby, even combined with aptamer modification technology, it is still a great challenge for enzyme/enzyme-like catalysts to realize precise and broad-spectrum sterilization.^4,9,10^ Taken together, an advanced diabetic wound treatment strategy should hold potential to break the above-mentioned limitations.

It's worth noting that the glucose enzymatic biofuel cell (GBFC), a special kind of biofuel cell, can generate electric energy *in situ* in bio-fluids *via* catalyzing glucose oxidation.^15–18^ And intriguingly, their anodic reaction catalyzed by glucose oxidase follows the path of glucose + O_2_→ gluconate + H_2_O_2_.^19,20^ Clearly, the GBFC is expected to take advantage of hyperglycemia in diabetic wounds to generate abundant H_2_O_2_. It's also worth noting that the surfaces of bacteria are known to be negatively charged in the biological environment. In particular, some bacteria, such as *Escherichia coli*, will produce electrons during their metabolism, so they prefer to adhere to the conductive interface.^21–23^ As a result, the electric field generated by the GBFC, in theory, is highly helpful in promoting the adhesion of bacteria on the electrode surface. Given this, precise sterilization is expected to be realized in a GBFC-powered system. Despite the charming application potential of GBFC-powered systems in managing diabetic wounds, no relevant research has been reported so far.

**Scheme 1:**
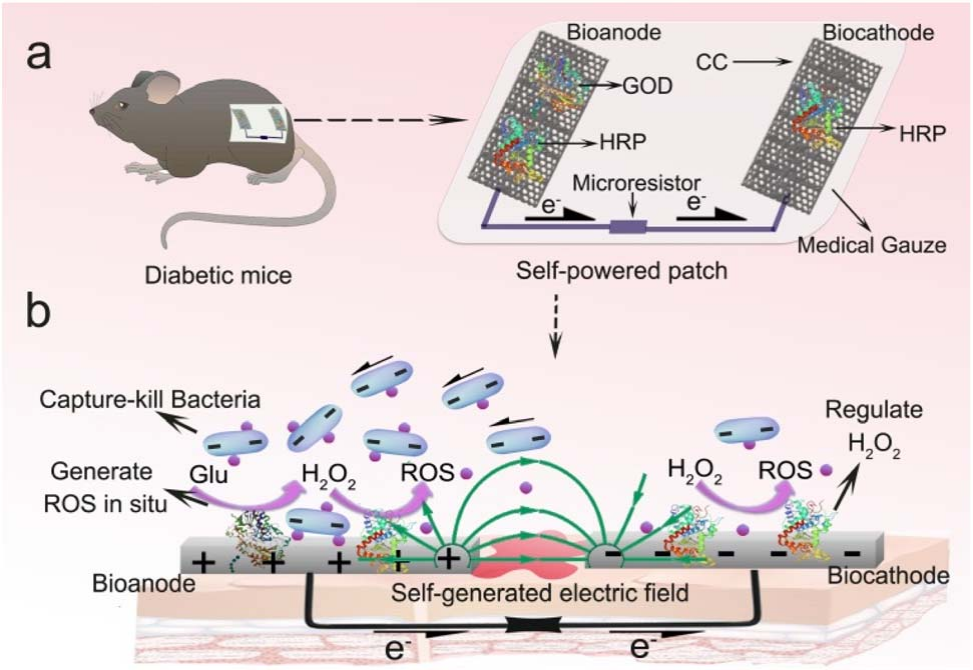
(a) The schematic representation of a GBFC-powered antibacterial patch for treating diabetic wounds. (b) The illustration of the working principle of this patch for regulating local glucose, generating abundant ROS *in situ*, and precisely sterilizing driven by an electric field.

Herein, we designed a GBFC-powered and drug-free antimicrobial patch for treating diabetic wounds. The core components of this patch were an MAF-7 protected glucose oxidase (GOD)/horseradish peroxidase (HRP) anode, a HRP cathode, and an external resistor. To design a flexible patch, flexible carbon cloth (CC) with outstanding electron conductivity was used as the support electrode (Scheme 1a). Systematic experiments proved that two distinctive features of this self-powered patch enhanced its function in accelerating diabetic wound healing. First, the MAF-7 protected GOD/HRP anode could successively catalyze the aerobic oxidation of glucose and the decomposition of H_2_O_2_, taking advantage of the hostile hyperglycemia to produce abundant antibacterial agents of ROS. And the HRP cathode exhibited high catalytic activity toward the decomposition of H_2_O_2_, and thus it could regulate the accumulation of H_2_O_2_ and effectively avoid the damage of H_2_O_2_ to normal tissues. In particular, the experimental results confirmed that the GBFC could create an electric field *in situ* in the wound environment, and drive the negatively charged bacteria to adhere firmly to the anode surface. As a result, the ROS generated on the electrode was localized to the bacteria, realizing precise sterilization (Scheme 1b). The promotion effect of the GBFC-powered antimicrobial patch on diabetic wound healing was investigated using model diabetic mice (Scheme 1). As a result of the synergistic effects of local glucose regulation, abundant ROS generation, and electrically driven precise sterilization, the inflammation caused by bacterial infection was rapidly eliminated. And the skin wounds of the diabetic mice treated with the GBFC-powered patch could be healed within 10 days, showed no inflammatory cell infiltration and formed mature skin with new hair follicles. It's believed that this contribution provides a new opportunity in designing a new-generation antibacterial patch for treating chronic wounds without using any exogenous drugs or adjuvants.

## Results and discussion

### Fabrication and characterization of a GBFC-powered patch

**Fig. 1:**
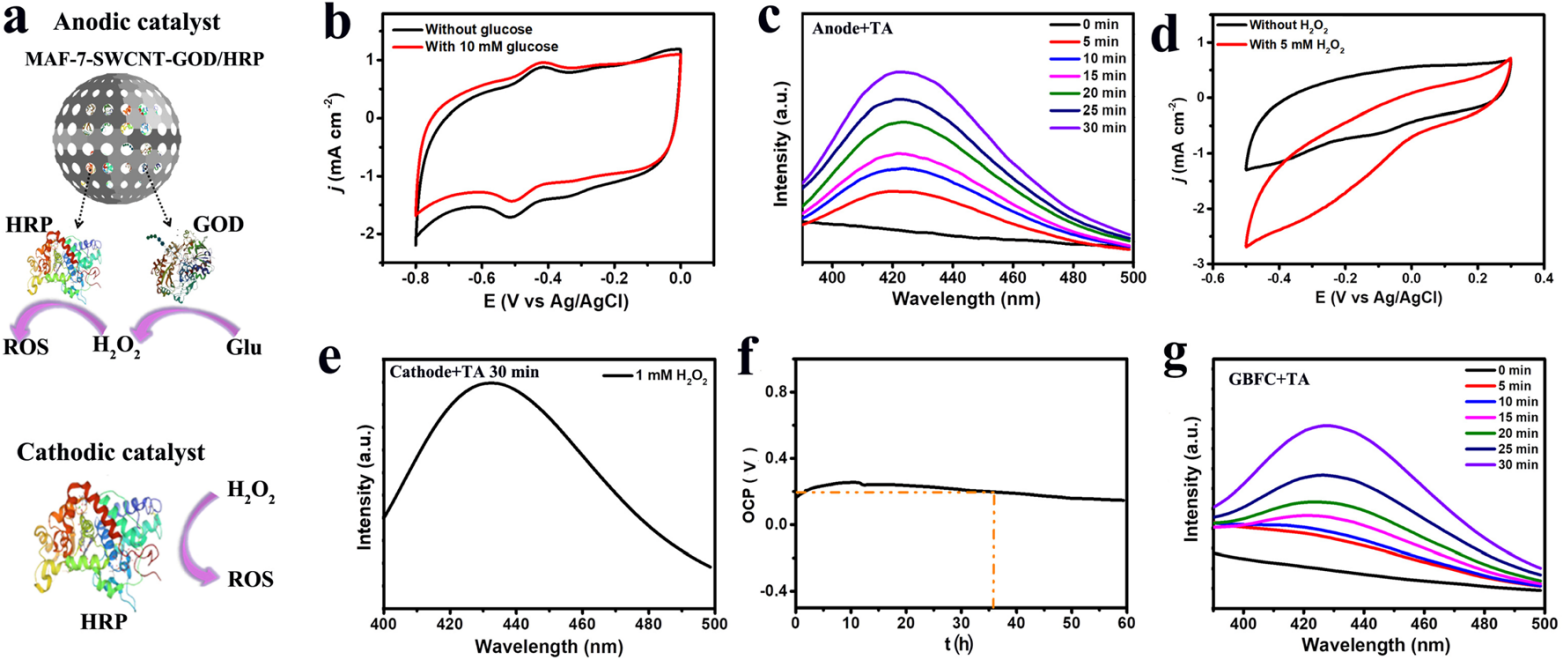
(a) The anodic and cathodic catalysts of the GBFC. (b) The CV curves of the MAF-7-SWCNT-GOD/HRP/CC anode with (red) and without (black) 10 mM glucose. Scan rate: 25 mV s^−1^. (c) The anode's OH˙ generation capacity in the presence of 10 mM glucose. The fluorescence emission spectra were collected after discharging the anode for different times. (d) The CV curves of the HRP/CC cathode with (red) and without (black) 5 mM H_2_O_2_. Scan rate: 25 mV s^−1^ (e) the cathode's OH˙ generation capacity in the presence of 1 mM H_2_O_2_. The fluorescence emission spectra were collected after discharging the cathode for 30 min. (f) The open circuit voltage curve of the GBFC in the blood of diabetic mice. (g) The GBFC's OH˙ generation capacity. The fluorescence emission spectra were collected after discharging the GBFC for different times. Discharge current: 20 μA cm^−2^.

This work was begun by designing a GBFC that can synergistically regulate the local glucose level and generate abundant ROS. Regarding the anode fabrication, we encapsulated the cascaded enzymes of GOD and HRP, and the electron nanowire of single-walled carbon nanotubes (SWCNTs) into MAF-7, because the metal–organic framework has been proven to be effective in protecting enzymes from biological corrosion.^19,24^ The scanning electron microscopy (SEM) and XRD results demonstrated the formation of spherical MAF-7-SWCNT-GOD/HRP (Fig. S1–S3†). Finally, the obtained MAF-7-SWCNT-GOD/HRP was coated on the flexible CC to serve as the anode (Fig. 1a). For a deeper study of the electro–catalytic activity of the obtained anode, we performed cyclic voltammetry (CV) tests. The MAF-7-SWCNT-GOD/HRP anode showed well-defined redox peaks at −0.4/−0.5 V, which were highly consistent with the theoretical redox potential of GOD (Fig. 1b).^17,19^ This clearly confirmed the successful encapsulation of GOD and the direct electron transfer between GOD and the electrode. It's also worth noting that the peak current significantly increased to 1.0 mA cm^−2^ from 0.6 mA cm^−2^ after adding 10 mM glucose, suggesting the high activity of the anode toward glucose oxidation.

Also, we examined the ROS generation capacity of the anode by a fluorescence test. Considering that the aerobic oxidation of glucose will generate H_2_O_2_ and HRP can decompose H_2_O_2_ into OH˙, therefore, in theory, the MAF-7-SWCNT-GOD/HRP anode could produce OH˙, a kind of ROS that can cause fatal injury to bacteria due to its super oxidability. To confirm this, we monitored the fluorescence change of the anolyte in the presence of terephthalic acid (TA), since OH˙ can oxidize TA into *o*-hydroxyterephthalic acid (HTA), which has a fluorescence emission peak at 420 nm.^12^ We noticed that a fluorescence emission peak appeared at 420 nm in the presence of glucose, and the peak intensity increased with the increase of the glucose level, whereas, no detectable fluorescence could be observed in the absence of glucose, confirming the impressive glucose-dependent OH˙ generation capacity of the anode (Fig. S4†). Furthermore, we also examined the OH˙ generation rate of the anode at a high glucose level (10 mM). Significantly, when the anode discharged at a current density of 20 μA cm^−2^, the fluorescence peak intensity rapidly increased to a high level within 30 min, signifying a remarkable OH˙ generation rate (Fig. 1c). Taken together, we could conclude that the MAF-7-SWCNT-GOD/HRP anode underwent the cascaded electrode reactions of glucose + O_2_ → gluconic acid + H_2_O_2_ → OH˙ (Scheme 1), and thus was able to achieve the synergistic functions of regulating local blood glucose and inhibiting bacterial infection.

With regard to the cathode design, the accumulation of H_2_O_2_ has been proven to be a main contributor to the decrease in immunity.^2^ Therefore, the cathode was expected to be able to eliminate the excess H_2_O_2_. With this in mind, we used HRP, which is known to be highly active in scavenging H_2_O_2_ to generate ROS, as the catalyst of the cathode.^25,26^ And the composite of SWCNT/AuNPs was used as the substrate material to accelerate the electron transfer and to provide abundant toeholds for HRP immobilization (Fig. S5†). As expected, the cathodic current density significantly increased to 2.8 mA cm^−2^ in the presence of H_2_O_2_, demonstrating the high electro-catalytic activity of the HRP cathode toward the decomposition of H_2_O_2_ (Fig. 1d). What's more, in the presence of 1 mM H_2_O_2_ and TA, we observed a strong fluorescence emission peak (420 nm) at the cathode after a short discharge time of 30 min at a current density of 20 μA cm^−2^, signifying the generation of OH˙ (Fig. 1e). Therefore, the cathode was confirmed to be able to synergistically regulate the accumulation of H_2_O_2_ and generate more antibacterial agents of OH˙.

Next, we explored the electricity generation performance of the GBFC equipped with the MAF-7-SWCNT-GOD/HRP/CC anode and the HRP/CC cathode. In blood, this GBFC could offer a high power density of ∼32.4 μW cm^−2^ (Fig. S6†). What's more, the open-circuit voltage (*V*_OCP_) stayed stable at ∼0.2 V for a long time of 36 h (Fig. 1f). In the absence of glucose, the maximum power of the GBFC was only 3.21 μW cm^−2^, suggesting that the electric power generated by the GBFC was mainly from the efficient catalysis of the electrode catalysts toward the fuels (Fig. S7†). In contrast, for the GBFC equipped with the unprotected SWCNT-GOD/HRP/CC anode and the HRP/CC cathode, the *V*_OCP_ rapidly decreased to 0.05 V within 2 h and the maximum power density was only 14.5 μW cm^−2^ (Fig. S8†). This signified the efficiency and the antifouling capacity of the GBFC was impressive due to the highly protective effect of MAF-7 on the enzymes.^15,19,27^ Also of note, when the GBFC discharged at a current density of 20 μA cm^−2^ in the presence of TA, we could observe an obvious fluorescence peak at 420 nm rapidly increasing over time (Fig. 1g). This strongly confirmed the impressive OH˙ generation capacity of the GBFC. Consequently, the great application potential of this GBFC in designing self-powered and drug-free antibacterial patches was convincingly confirmed.

**Fig. 2:**
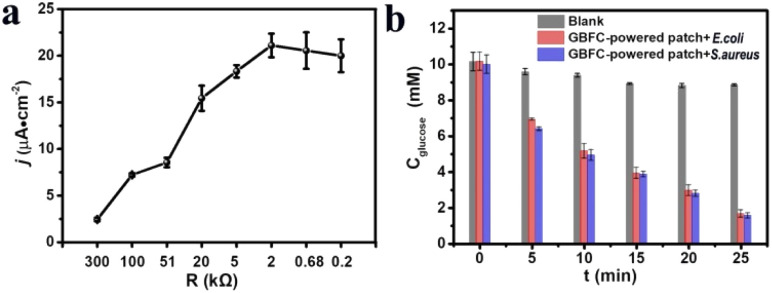
(a) The relation curve between the discharge current of the GBFC and the resistance value of the external resistor. (b) The glucose concentration in the bacterial culture media incubated with the GBFC-powered patch for different times.

Establishing a sufficiently high current *via* connecting a suitable external resistor is necessary for creating a practical GBFC-powered patch.^28–30^ So we investigated the relationship between the discharge current of the GBFC and the resistance value of the external resistor. The results revealed that the discharge current of the GBFC rapidly increased with the decrease of the external resistance value in a range of 300 kΩ to 2 kΩ. However, the current showed a slight decrease as the resistance value decreased further due to the large electrode polarization (Fig. 2a). Given this, a micro-resistor with a resistance value of 2 kΩ was chosen to connect the anode and the cathode. Eventually, the GBFC-powered antibacterial patch was assembled. Then, the glucose regulation capacity of this patch was studied carefully. As shown in Fig. 2b, when we placed this patch in the bacteria culture medium containing *Escherichia coli* (*E. coli*) or *Staphylococcus aureus* (*S. aureus*), the glucose concentration sharply decreased to ∼1.1 mM from 10 mM within 25 minutes, confirming the remarkable glucose-lowering properties of this patch (Fig. 2b).

### *In vitro* antibacterial effect of the GBFC-powered patch

**Fig. 3:**
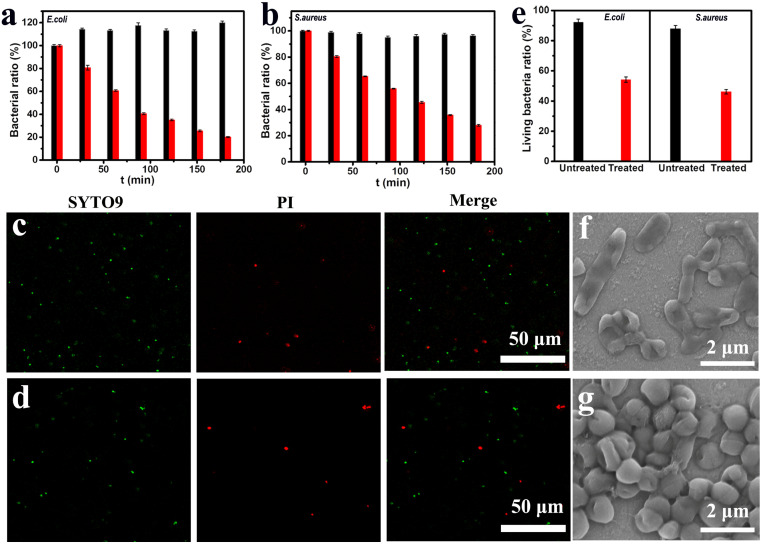
The bacterial density of the *E. coli* (a) and the *S. aureus* (b) in the culture medium incubated with (red column) and without (black column) the GBFC-powered patch. CLSM images of the *E. coli* (c) and *S. aureus* (d) collected from the bacteria culture medium incubated with the GBFC-powered patch for 3 h (green fluorescence: live bacteria stained with SYTO9 and red fluorescence: dead bacteria stained with propidium iodide (PI)). (e) Semiquantitative statistics of bacterial viability from analysis of the fluorescence intensity percent of SYTO9 over total fluorescence intensity of SYTO9 and PI. SEM images of the *E. coli* (f) and *S. aureus* (g) incubated with the GBFC-powered patch for 3 h.

The *in vitro* bactericidal effect of this patch was studied taking *E. coli* (Gram-negative) and *S. aureus* (Gram-positive) as model bacteria.^9,10,31^ For the *E. coli* without any treatment, their density in the culture medium exhibited a slight increase within 3 h. In contrast, for the *E. coli* incubated with the GBFC-powered patch, their density in the culture medium rapidly decreased by ∼80% (Fig. 3a and S9†). Similarly, in the case of *S. aureus*, the bacterial density also showed a high decrease rate of ∼70% after incubating with the GBFC-powered patch for 3 h (Fig. 3b and S10†). Next, we observed the viability of residual bacteria using confocal laser scanning microscopy (CLSM) *via* staining bacteria with a live/dead BacLight bacterial viability kit (green: living bacteria; red: dead bacteria).^6,9,13^ Both the *E. coli* and *S. aureus* incubated with the GBFC-powered patch showed strong red fluorescence, signifying a decreased bacterial viability (Fig. 3c and d). By analyzing the ratio of green fluorescence intensity, we found that the living bacteria ratio of *E. coli* and *S. aureus* decreased to 52% and 42%, respectively (Fig. 3e). Consistent with this, SEM tests revealed that the cell membranes of the residual *E. coli* and *S. aureus* were both ruptured (Fig. 3f and g). In contrast, for the bacteria treated with the inactivated enzyme modified patch, we observed no dead bacteria from the CLSM results, and the bacterial structure was undamaged, demonstrating the biosecurity of the patch's basic components (Fig. S11 and S12†). Since no exogenous drugs were added, we can conclude that the main cause of the destruction of the bacteria in the culture medium is due to the diffusion of the OH˙ generated on the electrode surface (Scheme 1b).

**Fig. 4:**
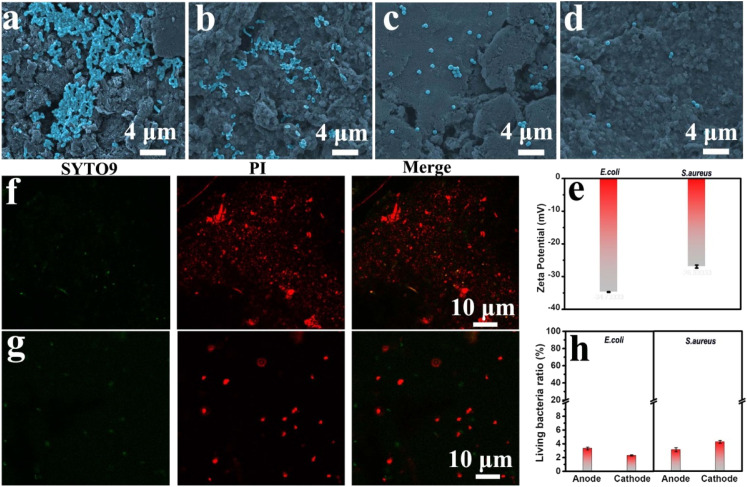
SEM images of the patch's electrodes incubated with *E. coli* (a) and (b) and *S. aureus* (c) and (b) for 3 h. (a) and (c) anode; (b) and (d) cathode. (e) Zeta potential of *E. coli* and *S. aureus*. The CLSM images of the anode of the GBFC-powered patch incubated with *E. coli* (f) and *S. aureus* (g) (green fluorescence: live bacteria stained with SYTO9 and red fluorescence: dead bacteria stained with propidium iodide (PI)). (h) Semiquantitative statistics of bacterial viability *via* analyzing the fluorescence intensity percent of SYTO 9 over the total fluorescence intensity of SYTO9 and PI.

Also of note, although the viability of the bacteria in the culture medium decreased, we could observe the bacteria with a ruptured cell membrane using SEM. That is, the bacteria were not completely broken within 3 h. However, as shown in Fig. 3a and b, the detectable bacteria in the culture medium significantly decreased. Therefore, figuring out where most of the bacteria went was crucial to reveal the antibacterial mechanism of this GBFC-powered patch. Given this, we observed the patch's electrodes that were exposed to the bacteria culture medium using SEM tests. For comparison, we first observed the disconnected anode and cathode incubated with the electro-active *E. coli* for 3 h. Some bacteria were observed on the anode and cathode (Fig. S13 and S14†). Since no electricity was generated in this case and the bacterial number on the anode and cathode had no significant difference, it's reasonable to assume that the adherent growth ability of bacteria was the main contributor to the adhesion of the bacteria on the electrode surface. Interestingly, in the case of the micro-resistor connected GBFC-powered patch, more *E. coli* were observed on the electrodes. In particular, the bacterial density on the anode was not only significantly higher than that on the cathode but, more importantly, far exceeded that on the anode of the disconnected patch (Fig. 4a and b). This demonstrated that the GBFC-powered patch could generate an electric field to drive the bacteria to adhere firmly to the electrode. The key reason for this was that *E. coli* were negatively charged and electro-active, and they preferred to adhere to the anode of the GBFC to smoothly release the self-produced electron (Fig. 4e).^22^ Even for the non-electroactive but negatively charged bacteria *S. aureus*, a higher bacterial density was also observed on the anode of the micro-resistor connected GBFC-powered patch, compared to that of the open-circuit patch (Fig. 4c and d). Taken together, the electric field generated by the GBFC was proven to be able to drive the bacteria to adhere firmly to the anode surface.

As we confirmed above, the GBFC-powered patch could produce abundant antimicrobials of OH˙ *in situ* on the electrode surface, and thereby the GBFC-powered patch was expected to achieve the goal of precise capture-sterilization. Clearly, as the SEM images revealed, the cell membranes of the overwhelming majority of bacteria captured by the electrodes were ruptured (Fig. 4c and d). To get stronger evidence, the bacterial viability on the electrode was analyzed using CLSM. The electrodes of the GBFC-powered patch incubated with *E. coli* or *S. aureus* both showed high density and strong red fluorescence and almost no green fluorescence was observed (Fig. 4f, g, S15 and S16†). By analyzing the green fluorescence intensity ratio, we found that the living bacteria ratio of *E. coli* and *S. aureus* both reduced to less than 5% (Fig. 4h). This strongly confirmed that the electric field generated by the GBFC-powered patch powerfully boosted the adhesion of bacteria on the electrode, and further localized the ROS generated on the electrode surface to the bacteria. As a result, the GBFC-powered patch achieved a highly effective and precise antibacterial performance.

In particular, although the bacterial density on the electrodes far exceeded that in the culture medium, the living bacteria ratio on the electrodes (<5%) was 10 times less than that in the culture medium (∼50%). This suggested that the ROS diffused into electrolyte from the electrode was limited. Therefore, apart from precise sterilization, the GBFC-powered patch could also avoid the indiscriminate attack of ROS on normal tissue cells.

### *In vivo* accelerated effect of the GBFC-powered patch on diabetic wound healing

**Fig. 5:**
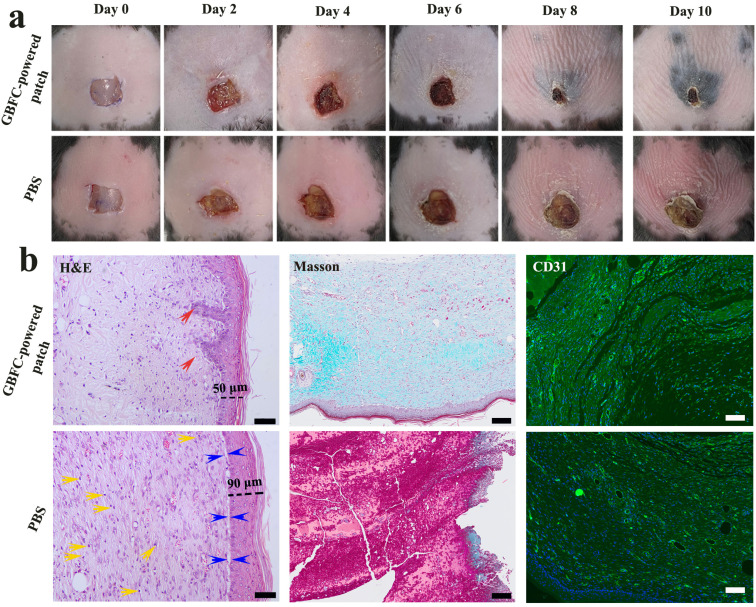
(a) Photographs of the *S. aureus*-infected wounds in diabetic mice treated with the GBFC-powered patch and PBS for different times. (b) Histologic analysis of the diabetic wounds after treating with the GBFC-powered patch and PBS for 10 days. H&E, Masson and CD31 staining were used to investigate the inflammation response, collagen deposition and neovascularization in the wound area, respectively. Scale bars: 50 μm (H&E), 100 μm (Masson), and 100 μm (CD31). In the H&E staining images, the red, blue and yellow arrows represent the nascent hair follicles, the space between dermis and subcutaneous tissue and the inflammatory cells, respectively.

Finally, the GBFC-powered antibacterial patch was applied to treat the *S. aureus*-infected skin wounds on the diabetic mice. For comparison, we first observed the natural healing process of diabetic wounds. Due to the weak immune system and hyperglycaemia, the diabetic wounds suffered from severe bacterial infection and a chronic healing process. Even after 10 days, the wounds treated with PBS showed a low closure rate of 35% (Fig. S17 and S18†). Significantly, the diabetic wounds treated with the GBFC-powered patch showed accelerated closure. An obvious wound closure was observed on the 2nd day, and the cut began to scab over 6 days, and in particular, the wounds dressed with the GBFC-powered patch achieved a high closure of 90% on day 10 (Fig. 5a). This confirmed the outstanding promoting effect of the GBFC-powered patch on diabetic wound healing.

For a deeper understanding of the wound healing process, the inflammation response, collagen deposition and neovascularization during the wound healing process were investigated by H&E, Masson and CD31 staining, respectively. For the skin wounds treated with PBS for 3 days, we observed a large area of inflammatory cell infiltration from the H&E staining results (Fig. S19†), signifying the severe bacterial infection in the diabetic wounds.^10,13,30^ In addition, the extracellular interstitium was disordered, and neither collagen deposition (Masson) nor neovascularization (CD31) could be observed, suggesting that the collagenous fiber suffered severe damage due to bacterial infection (Fig. S19†). For the diabetic wounds dressed with the GBFC-powered patch, although the diabetic wounds also showed an inflammation response after 3 days, we noted that partially ordered extracellular interstitium, nascent collagen fibers (Masson, blue), and blood vessels (CD31, green) started to be generated.^9,14,30,31^ This suggested that the GBFC-powered patch could have a rapid therapeutic effect to accelerate neovascularization (Fig. S19†).

After 10 days, compared to the wounds treated with PBS, the wounds treated with the GBFC-powered patch showed minor inflammatory infiltration, confirming the highly effective bacteriostatic function *in vivo* of the GBFC-powered patch.^32^ Meanwhile, a multilayered and close-connected skin tissue (thickness: ∼50 μm) with nascent hair follicles formed in the GBFC-powered patch treated group. In contrast, in the PBS group, the newly formed skin was thicker (thickness: ∼90 μm) and was not fully connected to the subcutaneous tissue. Meanwhile, we observed no nascent hair follicles on the healed skin. What's more, as the Masson and CD31 staining results revealed, more extensive and ordered collagen disposition and more neovascularization were observed in the GBFC-powered patch treated group compared to the group treated with PBS (Fig. 5b). This validated that the GBFC-powered patch could accelerate neovascularization during the wound healing process. As a result, a conclusion can be drawn that the GBFC-powered patch can effectively accelerate diabetic wound healing *in vivo via* breaking hyperglycemia and low H_2_O_2_ limitations and precisely sterilizing driven by electricity.

## Conclusions

In summary, we designed and validated a self-powered and drug-free antibacterial patch based on a GBFC for accelerating diabetic skin wound healing. This GBFC-powered patch consisted of a flexible MAF-7 protected GOD/HRP/CC anode and flexible HRP/CC cathode. This patch had three distinctive features: on the one hand, this patch could take advantage of hyperglycemia to generate abundant H_2_O_2_, breaking the limitations of high blood glucose levels and low H_2_O_2_ in diabetic wounds. On the other hand, this patch could further efficiently catalyze the decomposition of H_2_O_2_, generating abundant antibacterial agents of ROS *in situ* and reducing the damage the accumulated H_2_O_2_ does to the immune system. Thirdly and more importantly, this GFBC-powered patch could generate an electric field in the wounds to drive the negatively charged bacteria to adhere firmly to the anode's surface, eventually localizing the ROS generated on the electrode surface to the bacteria and avoiding the indiscriminate attack of ROS on the normal tissue cells. As a result, the *in vitro* antibacterial rate of this self-powered patch reached up to 95% within 180 min. And the bacteria-infected diabetic wounds treated with this GBFC-powered patch eliminated the inflammatory infiltrates within 10 days. In particular, one could observe new and close-connected skin tissue with nascent hair follicles, more extensive collagen disposition and more neovascularization in the healed wounds on diabetic mice. It is believed that this self-powered patch will be a promising new-generation and drug-free antibacterial patch in managing diabetes-related chronic wounds.

## Data availability

All data generated during this study are available either in the main text or ESI.†

## Author contributions

L. Wang and Q. Su designed the experiments and analyzed the data. Q. Su, Y. Liu and D. Hu performed the fabrication and biological experiments. All authors made contributions to discuss the results and comment on the manuscript. L. Wang and Q. Su interpreted the data. L. Wang wrote the original manuscript with important input from all other authors, L. Wang, J. Zhu and J. Zhang revised and finalized the manuscript. J. Zhu and J. Zhang supervised this work.

## Conflicts of interest

There are no conflicts to declare.

## Supplementary Material

SC-013-D2SC04242H-s001
