# Supplementary material for: A self-powered and drug-free diabetic wound healing patch breaking hyperglycemia and low H2O2 limitations and precisely sterilizing driven by electricity
Source: Chem Sci. 2022 Sep 30;13(41):12136–43. doi: 10.1039/d2sc04242h (PMC9601455; doi:10.1039/d2sc04242h)
Supplement: SC-013-D2SC04242H-s001 [file SC-013-D2SC04242H-s001.pdf]

Electronic Supplementary Material (ESI) for Chemical Science.
This journal is © The Royal Society of Chemistry 2022

Supporting Information

A self-powered and drug-free diabetic wound healing patch breaking hyperglycemia and low H_2O_2 limitations and precisely sterilizing driven by electricity

Linlin Wang,[a,b] Qiwen Su,[b] Yi Liu,[b] Tajiguli Yimamumaimaiti,[b] Dandan Hu,[b] Jun-Jie Zhu,[b*] Jian-Rong Zhang[b*]

[a]*Shaanxi Key Laboratory of Chemical Additives for Industry, College of Chemistry and Chemical Engineering, Shaanxi University of Science and Technology, Xi'an 710021, China.*

[b]*State Key Laboratory of Analytical Chemistry for Life Science, School of Chemistry and Chemical Engineering, Nanjing University, Nanjing 210023, P. R. China. Email: jjzhu@nju.edu.cn; jrzhang@nju.edu.cn.*

EXPERIMENTAL SECTION

Materials and Chemicals

E. coli K-12 (ATCC 29181) was purchased from American Type Culture Collection (Manassas, VA, USA). *S.aureus* (ATCC 25923) was purchased from Beijing Solarbio Science Technology Co. Glucose oxidase (GOx, E.C. 1.1.3.4, from Aspergillus niger), Horseradish peroxidase (HRP), 1-ethyl-3-(3-dimethyl-aminopropyl) carbodiimide hydrochloride (EDC) and N-hydroxysuccinimide (NHS) were obtained from Aladdin Company (Shanghai, China). Hydrogen peroxide solution(30%$_{vol}$, H_2O_2), disodium hydrogen phosphate dodecahydrate ($Na_2HPO_4 \cdot 12H_2O$), sodium Dihydrogen Phosphate Dihydrate ($NaH_2PO_4 \cdot 2H_2O$)，potassium dihydrogen phosphate(KH_2PO_4), sodium chloride(NaCl), terephthalic acid(TA), magnesium sulfate($MgSO_4$), polyvinylpyrrolidone (PVP, K-30), glucose, gold chloride($HAuCl_4$) and poly(diallyl dimethylammonium) chloride (PDDA) were purchased from Sinopharm Chemical Reagent Co. High purity Single Walled Carbon nanotubes (SWCNT) was purchased from Suzhou Tanfeng Tech Reagent Co. 3-methyl-1,2,4-triazole (Hmtz) was manufactured by Shanghai shaoyuan Co. Lid. Zinc nitrate hexahydrate ($Zn (NO_3)_2 \cdot 6H_2O$) was obtained from Xilong Chemical industry Co. LTD. uria-Bertani (LB) broth was obtained from Sigma Aldrich. PBS solution (100 mM, pH=7.4) consisting of Na_2HPO_4 and NaH_2PO_4 and M9 buffer solution (KH_2PO_4, 3 mg mL^{-1}; $Na_2HPO_4 \cdot 12H_2O$, 15 mg mL^{-1}; NaCl, 5 mg mL^{-1}; $MgSO_4$, 0.12 mg mL^{-1}) were prepared for further use. Detergent Compatible Bradford Protein Assay Kit (Bradford Kit) was purchased from Beyotime Biotechnology. LIVE/DEAD BacLight Bacterial Viability Kit was obtained from Thermo Fisher Scientific (China) Co., Ltd. Ultrapure water (18.2 MΩ resistivity, Milli-Q, Millipore) was used in all experiments. All media and solutions were sterilized before use. 0.1 M pH=7.4 phosphate buffer solution (PBS) consisting of Na_2HPO_4 and NaH_2PO_4 was employed as the supporting electrolyte.

All kinds of resistances and wire were purchased from Shenzhen Kebi Micro Semiconductor Co., Ltd. Release paper film and pressure sensitive tape were purchased from Qingdao Hainuo Bioengineering Co., Ltd.

Apparatus

Zeta potentials were measured by ZETASIZER nanoseries (Nano-ZS, Malvern) at room temperature. X-ray diffraction (XRD) measurements were conducted on a D8 ADVANCE X-ray diffractometer (Bruker Corporation, America). Fluorescence spectra were recorded on a F-7000 (HITACHI, Japan). Scanning electron microscopy (SEM) images was obtained by JSM-7800F, JEOL, Japan. OD of bacteria was measured by full wavelength reader (ThermoFisher Varioskan Flash , America). CLSM studies were performed using a Leica TCS SP8 microscope (Germany). All electrochemical measurements were performed using an electrochemical workstation (CHI660D, Chenhua, China) in a three-electrode system consist of platinum wire counter electrode, Ag/AgCl reference electrode and working electrode.

Pretreatment of the carbon cloth

In this study, the flexible and electron-conductive carbon cloth (CC) was used as the substrate electrode to support the anode's and cathode's enzymatic catalysts. Firstly, the CC was cut into a small size of 0.5*1.5 cm. To eliminate the bio-toxicity of the CC caused by the surface contamination, we washed the CC with $75\%_{vol}$ ethanol and deionized water for three times, respectively, and then sterilized the CC with an autoclave. In addition, to increase the CC's surface hydrophilicity to create an effective bio-interface, the dried CC was further treated by oxygen plasma for 120 s.

Preparation of the anode

The anode's catalyst was fabricated according to the method we reported previously. 30 mg of PVP and 4 mg of SWCNT were dispersed into 0.5 mL deionized water, and then the mixture were treated by successively ultrasonic processing and mechanical agitation for 30 min and 2 h, respectively. As a result, we obtained a highly dispersed SWCNT ink. Next, 0.5 mL of solution containing 0.15 M Hmtz, 8 mg GOD, and 2 mg HRP were added to the SWCNT ink. After stirring for 30 min, 40 µL of 25% $NH_3{\cdot}H_2O$ containing 0.08 M $Zn(NO_3)_2{\cdot}6H_2O$ as added. Finally, the black precipitate appeared after stirring for 30 min and resting for 24 h at room temperature. Washing

thoroughly the precipitate with water, and then diluting the precipitate to 1 mL, we got the anode's catalyst solution of MAF-7-SWCNT-GOD/HRP which encapsulated the cascaded enzyme of GOD and HRP. The solution was stored at 4 ^{o}C for further use.

The anode was fabricated by dropping 40 μL of the MAF-7-SWCNT-GOD/HRP solution onto the pre-treated CC and dried at room temperature.

To study the protective effect of MAF-7 on the enzyme, we fabricated the SWCNT-GOD/HRP anode and analyzed the performance of the GBFC equipped with the SWCNT-GOD/HRP anode. The SWCNT with carboxyl was selected the electron conductive substrate to fix enzyme via amide condensation. Specifically, a 4 mg mL^{-1} SWCNT solution was dropped on the surface of the carbon cloth. Then, the electrode was dried at 37 °C for 2 h and immersed in a solution containing EDC/NHS (1 mg mL $^{-1}$) for 30 min to activate the carboxyl group. And then the activated electrode was incubated with the GOD and HRP solution (8 mg mL^{-1} of GOD and 2 mg mL^{-1} of HRP) for 12 h at 4 °C. Finally, the surface of the electrode was rinsed with deionized water and ready for use.

Preparation of the cathode

The cathode's catalyst we selected was HRP which could catalyze the decomposition of H_2O_2 to generate ROS. To accelerate the electron transfer rate between the HRP and electrode, as well as provide HRP with sufficient fixed sites, a composite consisted of SWCNT and AuNPs with carboxyl groups was firstly fabricated via electrostatic adsorption. In brief, to fabricate AuNPs with carboxyl groups, 96 mL of ultrapure water was added into 4 mL of 1% $HAuCl_4$ solution, and the mixed solution was heated with stirring. After the solution was boiling, 10 mL of 38.8 mM sodium citrate was quickly added into the solution and the reaction system was stirred vigorously under boiling conditions. After reaction for 15 min, the solution color began to change from pale yellow to wine-red, after cooling to temperature, we obtained the AuNPs solution. Then we dispersed 5 mg of SWCNT in the solution of 5 mL 1% PDDA, and treated the mixture by ultrasonic processing for 30 min, followed

by centrifugation and rinsing with deionized water. Finally, we got the positivity-charged SWCNT, which was re-dispersed into 100 mL solution containing negatively-charged AuNPs. The negatively-charged AuNPs could be absorbed by the positivity-charged SWCNT under stirring for 12 h, followed by centrifugation to remove the excessive AuNPs. The resultant SWCNT/AuNPs was lyophilized, followed by repeatedly grinding in 15_{wt}% polytetrafluoroethylene emulsion. The homogenous SWCNT/AuNPs paste was rolled out into a thin sheet, which can be cut into desired size and pressed onto carbon cloth. The mass load of the SWCNT/AuNPs was 2 mg cm^{-2}.

To fix the HRP onto SWCNT/AuNPs/CC, we firstly activated the carboxyl group on AuNPs by incubating the SWCNT/AuNPs/CC electrode with the solution of EDC/NHS (1 mg mL^{-1}) for 30 min. Next, the activated SWCNT/AuNPs/CC electrode was incubated with 4 mg mL^{-1} HRP for 12 h, followed by rinsing with water. The resultant HRP/SWCNT/AuNPs /CC was stored in 4 °C and ready for use.

Assembly of the GBFC-powered antibacterial patch

The MAF-7-SWCNT-GOD/HRP anode and the SWCNT/AuNPs/HRP cathode were pasted onto the flexible medical gauze using electrically conductive adhesives. To create a complete circuit, a tiny resistor was connected between the anode and cathode. In order to optimize the resistance value to ensure that the self-powered patch could offer relatively high electric energy, the currents of the GBFC-powered patches connected by resistors with different resistance value were measured using a multimeter. The resistance values were set to 0.2 kΩ, 0.68 kΩ, 2 kΩ, 5 kΩ, 20 kΩ, 50 kΩ, 100 kΩ, and 300 kΩ.

Determination of the enzyme loadings in MAF-7-SWCNT-GOD/HRP

The enzyme loading in MAF-7-SWCNT-GOD/HRP was investigated using BCA protein assay kit which could stain protein with a characteristic absorbance at 595 nm. Firstly, the standard BSA solution with certain concentrations of 0.125、0.25、0.5、0.75、1、1.5 mg mL^{-1} were prepared and incubated with the BCA kits for 30 min, followed

by the measurement of the absorbance at 595 nm (A_{595}). From the above results, we could establish the standard curve of protein concentration versus A_{595}. Then the resultant MAF-7-SWCNT-GOD/HRP solution was incubated with the BCA kit for 30 min, followed by centrifugation to collect the supernatant. To avoid the absorbance beyond the linear range, the absorbance of the supernatant was measured after 4-fold dilution. Finally, the amount of residual enzyme was calculated according to the standard curve. The enzyme loading in MAF-7-SWCNT-GOD/HRP could be estimated by subtracting the amount of residual enzyme.

CV measurement

Cyclic voltammograms were conducted in a three-electrode system with the CHI 660E electrochemical work-station using Pt wire as counter electrode, Ag/AgCl as reference electrode, and the MAF-7-SWCNT-GOD/HRP anode or SWCNT/AuNPs/HRP cathode as working electrode.

The electrochemical performance of the GBFC in the blood of diabetic mice

In practical application, the performance of the GBFC was the guarantee of creating a self-powered patch with efficient and stable antibacterial ability. So we firstly investigated the stability of GBFC by monitoring its open-circuit potential using the CHI 660E electrochemical work-station. Then, we recorded the polarization curves of the GBFC from the open-circuit potential to 0.0 V, and calculated the power density the GBFC could be offered based on the polarization curve using the formula of P=UI.

The OH• generation capacity of the anode, cathode, and the GBFC-powered patch

It has been proven that the OH• can oxidize terephthalic acid (TA) into o-hydroxyterephthalic acid (HTA), which has fluorescence characteristic peak at 420 nm. Therefore, we explored the OH• generation capacity of the anode, cathode, and the GBFC-powered patch via adding TA (final concentration 50 μM) to these systems, and recorded the fluorescence spectra in a range of 390 nm-500 nm (Ex: 315 nm) after incubating with the TA for certain time.

With regard to the anode, in presence of 50 μM TA, we discharged the anode

(discharge current: 20 μA cm^{-2}) in the electrolyte containing different concentrations of glucose for 30 min (glucose concentration: 0 mM, 5 mM, 10 mM, 15 mM) and recorded the fluorescence spectra of the anolyte to investigate the glucose-dependent OH• generation capacity of the anode. From the above experiments, we could only validate the effect of glucose on the OH• generation capacity of the anode. However, the MAF-7-SWCNT-GOD/HRP anode immobilized two enzymes of GOD and HRP, therefore, the anode was expected to display cascaded catalytic activity toward the reaction of $glucose + O_2 \xrightarrow{GOD} Gluconic\ acid + H_2O_2 \xrightarrow{HRP} OH\cdot$. To confirm this, we also recorded the fluorescence spectra of the anolyte in presence of 0.5 mM H_2O_2 after discharging for 30 min. In addition, to explore the OH• generation rate of the anode, we discharged the anode (discharge current: 20 μA cm^{-2}) in the electrolyte containing 10 mM glucose and 50 μM TA, and recorded the fluorescence spectra of the anolyte at different discharge time.

To investigate the OH• generation capacity of the cathode, we discharged the cathode (discharge current: 20 μA cm^{-2}) in the catholyte containing 50 μM TA and 1 mM H_2O_2 for 30 min, and recorded the fluorescence spectra of the catholyte at this point.

A tiny resistor with resistance value of 2 kΩ was proven to be able to enable the GBFC-powered patch to offer the highest current. Therefore, the final patch consisted of MAF-7-SWCNT-GOD/HRP anode, SWCNT/AuNPs/HRP cathode, and a tiny resistor with resistance value of 2 kΩ. We operated this patch in the electrolyte containing 50 μM TA. Subsequently, we recorded the fluorescence spectra of the electrolyte at different operation time.

Bacteria culture

The *E.coli* and *S. aureus* were cultured in LB broth at 37 °C. After reaching logarithmic phase, the bacteria were dispersed in the M9 buffer containing 10 mM glucose. And the bacterial density was determined using the absorbance at 600 nm (OD_{600}). The bacteria with OD_{600} value of 0.25 was set as the initial densities.

SYTO9/PI two-color fluorescent imaging

5 μL SYTO9 solution and 15 μL PI solution were added to 5 mL of 0.1 M PBS and mixed thoroughly to obtain the staining solution. The bacteria solutions that had been treated with the anode, cathode, and GBFC-powered patch were centrifuged to remove the supernatant, and washed with 0.1 M PBS three times. Subsequently, the obtained bacteria were re-dispersed in 200 μL PBS (0.1 M, pH 7.0), followed by the addition of 10 μL SYTO9/PI staining solution. After reacting for 20 min at 37 °C under dark condition and removing the excessive dye via centrifugation, we observed the bacterial state using Leica SP8 STED 3X confocal laser-scanning microscope (CLSM). (SYTO9: Ex 488 nm, Em 543 nm; PI: Ex 543 nm, Em 605 nm)

To analyze the bacterial state captured on the electrodes, we thoroughly washed the electrodes using PBS (0.1 M, pH 7.0) and incubated them with 10 μL SYTO9/PI staining solution for 30 min at 37 °C under dark condition, followed by washing using PBS. Finally, the stained electrodes were observed using Leica SP8 STED 3X confocal laser-scanning microscope (CLSM).

In addition, we also investigated the morphology of the treated bacteria using SEM. Specifically, the treated bacterial solution was centrifuged to remove the supernatant, followed by washing using PBS. Subsequently, the bacteria were re-dispersed in glutaraldehyde solution (2.5_{vol}%) and reacted for overnight at 4 °C, followed by centrifugation to remove the excessive glutaraldehyde. And then, the bacteria were successively treated in ethanol solutions with different volume concentrations (25%, 50%, 75%, 95%, and 100%) for 30 min following the above steps for dehydration. Finally, the obtained bacteria were observed using SEM. The bacteria on electrode were also treated by glutaraldehyde and ethanol following the above steps, and then observed using SEM.

The *in vitro* hyperglycemia regulation ability of the GBFC-powered patch

The hyperglycemia in diabetic wounds was one of the underlying causes of bacterial infection and long healing time. Given this, we investigated the glucose-lowering capacity of this resistor-connected patch via incubating the patch with bacterial

solutions (*E.coli* and *S. aureus*) containing 10 mM glucose and recording the glucose concentration using glucometer at different time.

In vitro antibacterial performance of the self-powered patch

This patch performed the antibacterial effect depend solely on the GBFC which could generate sufficient OH• using high concentration glucose and O_2 as fuels. Therefore, the antibacterial effect of this patch could be investigated just by incubating the bacteria solution (5 mL, OD_{600}) with the GBFC-powered patch for 3 h in presence of 10 mM glucose under air. Subsequently, we examined the final bacterial density using ultra-violet absorption spectrometry and characterized the bacterial activity by SYTO9/PI fluorescent double staining method.

For comparison, we also designed blank and control experiments. In blank group, the bacteria were cultured in M9 buffer containing 10 mM glucose at 37 ℃ for 3 h without any treatment. To explore the bio toxicity of the patch’s elements (such as carbon cloth, medical gauze, and electrode materials) to the bacteria, we deactivated the patch’s anode and cathode at 80 ° C, followed by incubating the patch with bacteria (5 mL, OD_{600}) for 3 h. Finally, we observed the bacterial morphology using SEM and analyzed their activity by SYTO9/PI fluorescent double staining method.

In vivo therapeutic effect of the self-powered patch on _S. aureus_ -infected diabetic wounds

To study the therapeutic effect of the GBFC-powered patch on actual diabetic wounds, we designed a series of animal experiments. The BKS.DB diabetic mice (age: 8 weeks, male) bought from Nanjing Zhonghua Biotechnology Co., Ltd were used as research models. We cut a wound with the size of 1 cm*1 cm on the back of every mouse. To create *S. aureus*-infected wounds, we sprayed 40 μL 1×10^7 CFU *S. aureus* solution on each wound. Subsequently, the wounds treatments were carried via wrapping the wounds with the GBFC-powered patch, and replaced a new GBFC-powered patch every 48 h. In order to ensure that the fuels in the blood can contact

with the electrode sufficiently, we dropped 100 μL PBS solutions onto the GBFC-powered patch every 4 h. In control groups, the medical bandages without the GBFC-powered patch were used. We monitored the changes in wounds within 10 days.

Histological analysis

After treating for 3-day and 10-day, we treated the mice using CO_2 to sacrifice them, and then dissected them. To obtain rigorous results, we conducted four-parallel experiments for each lab. The wound tissues were placed into 4% paraformaldehyde for 48 h to be degenerated and coagulated, followed by thoroughly washing using water for 30 min and dehydrating in different concentrations of ethanol (75%-4 h, 58%-2 h, 90%-2 h, 96%- 4 h, 100%- 45 min). Then, the tissues were put into melted paraffin. After cooling, they were sliced using microtome, and the thickness of the slice did not exceed 3 μm. To stain the tissue slice, we flatten them using biological tissue stall baking machine, followed by incubating with dyes of hematoxylin and eosin for 5 min and 1 min, respectively. Finally, we washed the slice using water to remove the excessive dyes, and observed the sections under a digital microscope (Nikon Eclipse Ci, Japan).

Supporting Figures

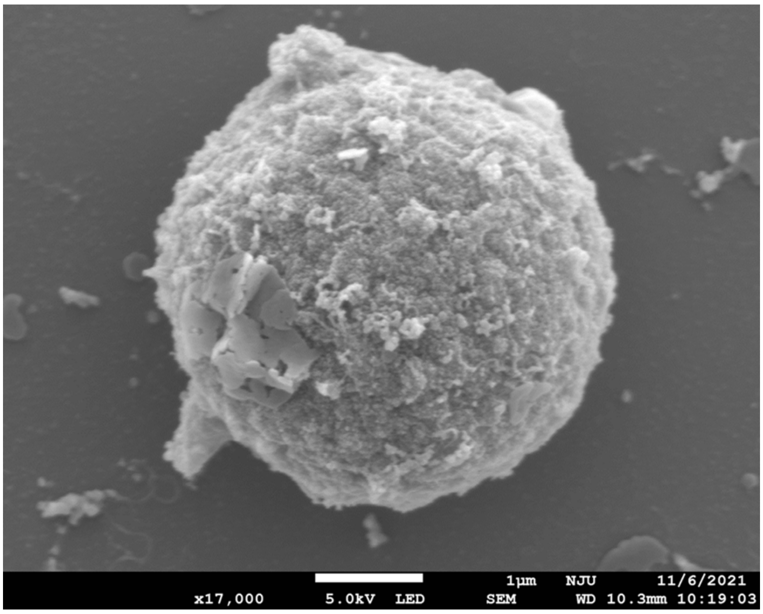

Figure S1. The SEM image of MAF-7-SWCNT-GOD/HRP.

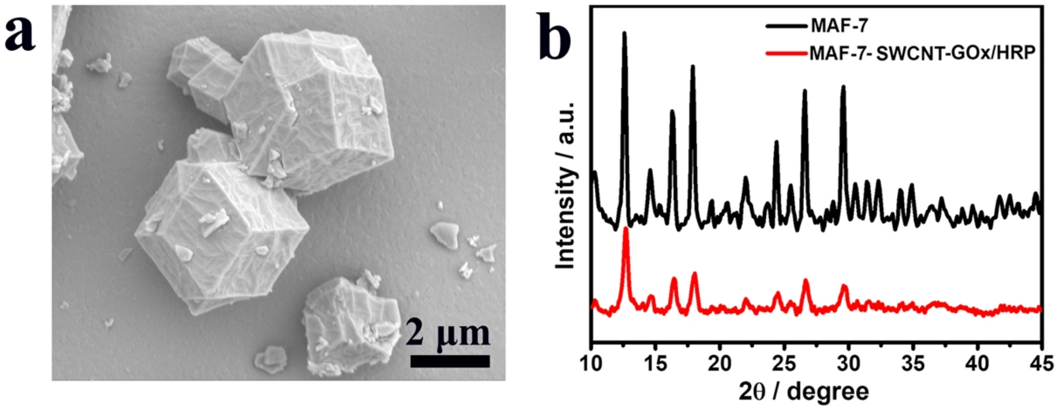

Figure S2. (a) SEM image of MAF-7. (b) XRD pattern of the MAF-7 and the MAF-7-SWCNT-GOD/HRP.

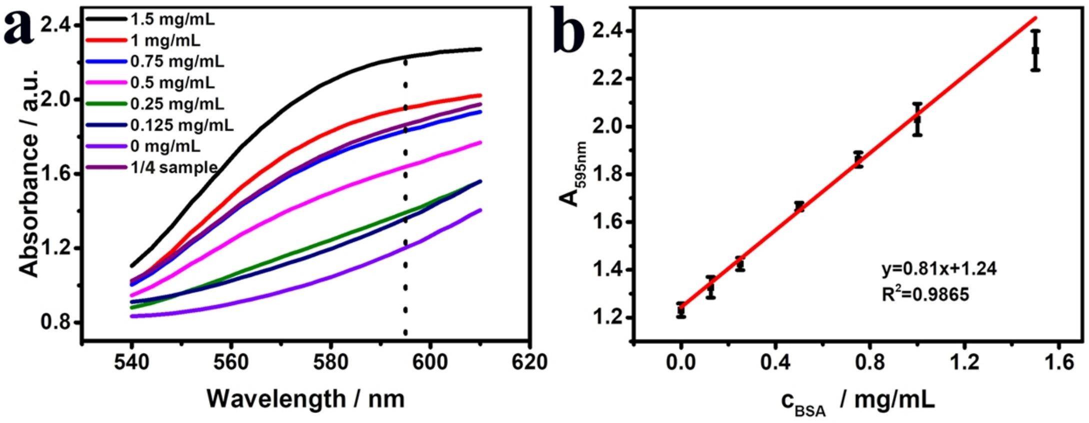

Figure S3. (a) The UV-vis absorption curves of the BSA with different concentrations and (b) the corresponding linear fitting curve of the absorbance at 595 nm versus the BSA concentration.

The UV-vis results revealed that the absorbance at 595 nm ($A_{595\ nm}$) and the protein (BSA) concentration followed a linear relationship of $A_{590nm} = 0.81C_{BSA}+1.24$ (R^2=0.9856). To estimate the enzyme loading encapsulated in the MAF-SWCNT-GOD/HRP, we collected the supernatant after the fabrication and recorded the UV-vis spectrum. The concentration of the residual enzyme caculated according to the standard curve was 3.2 mg mL^{-1}. Since 5 g of MAF-SWCNT-GOD/HRP was fabricated finally, the enzyme loading in MAF-SWCNT-GOD/HRP was eatibmated to 1.7 mg g^{-1}.

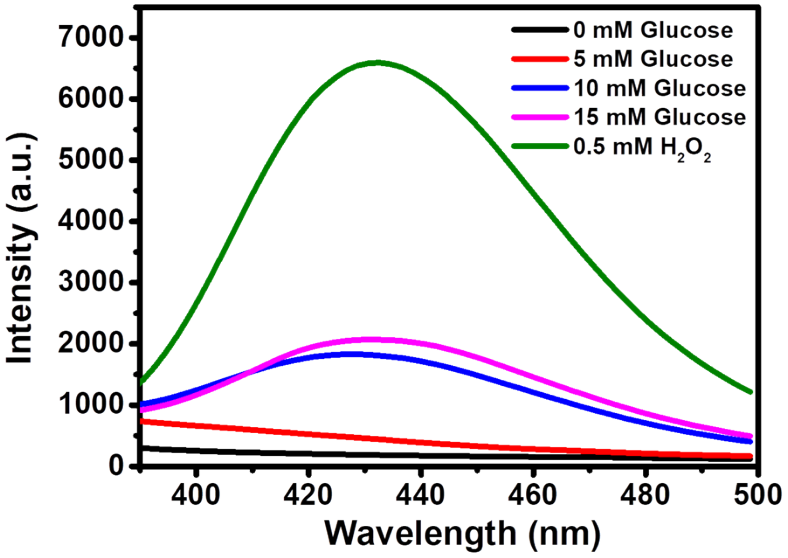

Figure S4. The OH· generation capacity of the anode in presence different concentrations of glucose and 0.5 mM H_2O_2. The fluorescence emission spectrums were collected after discharging the anode for 30 min. Discharge current: 20 μA cm^{-2}.

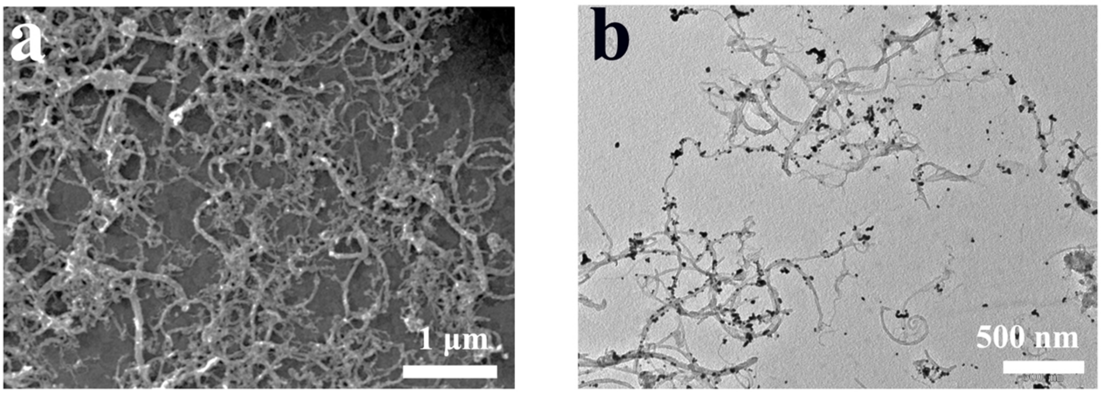

Figure S5. The SEM (a) and TEM (b) images of the SWCNT/AuNPs.

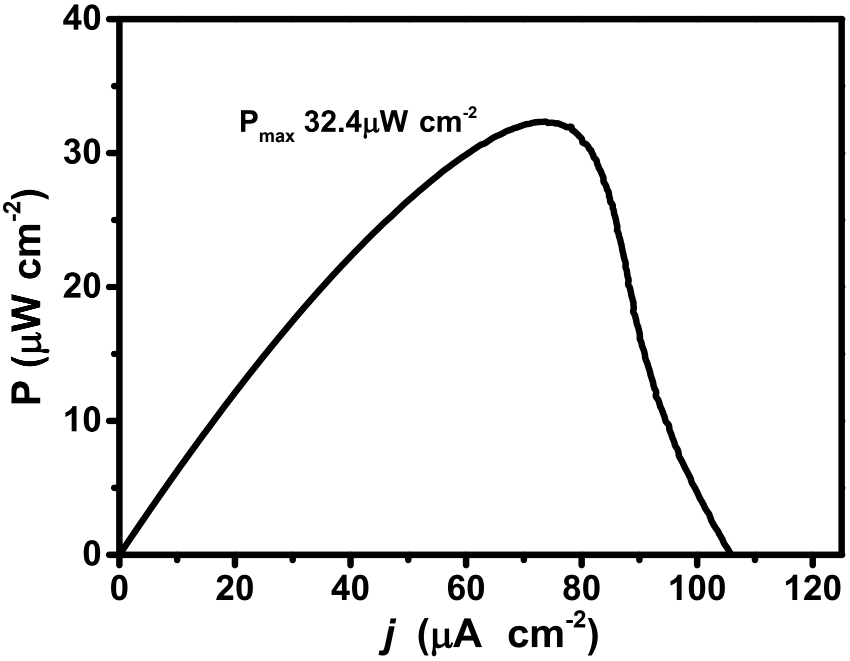

Figure S6. The power density output curve of the GBFC equipped with the MAF-7-SWCNT-GOD/HRP anode and the HRP cathode in the blood of diabetic mice. Scan rate: 10 mV s^{-1}.

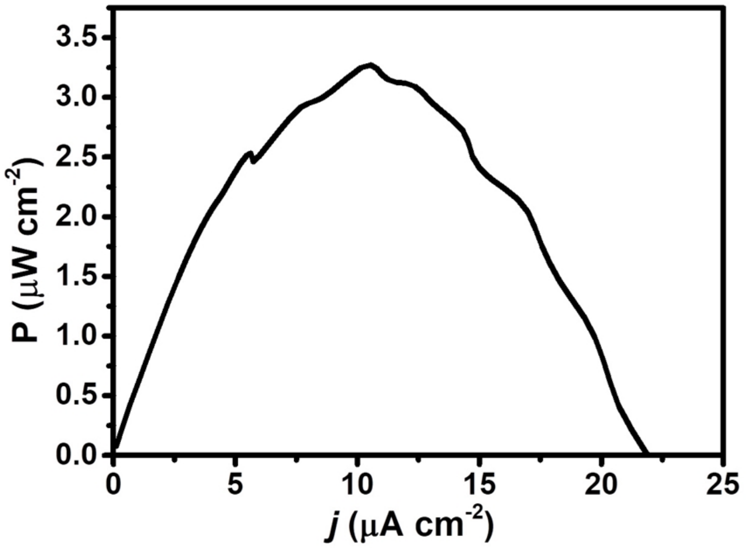

Figure S7. The power density output curve of the GBFC equipped with the MAF-7-SWCNT-GOD/HRP anode and the HRP cathode in the absence of glucose.

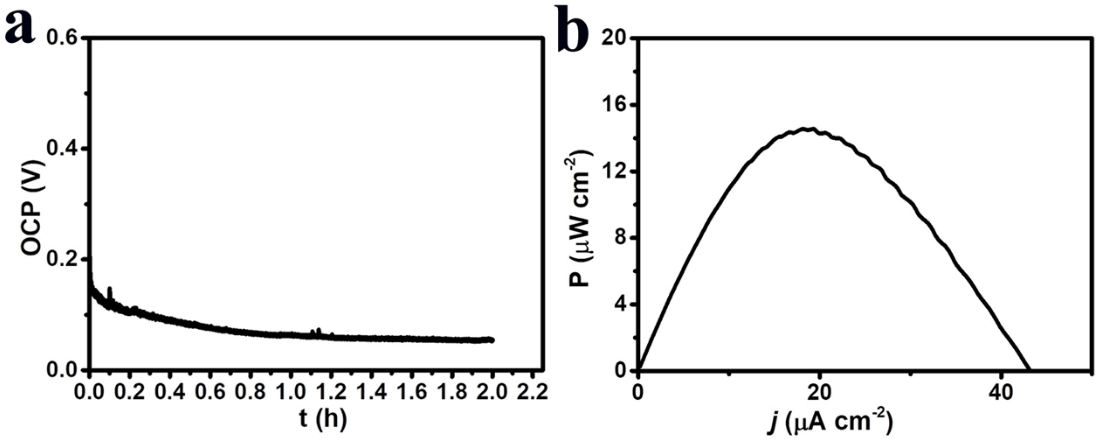

Figure S8. The open circuit voltage curve (a) and the power density output curve (b) of the GBFC equipped with the SWCNT-GOD/HRP anode and the HRP cathode.

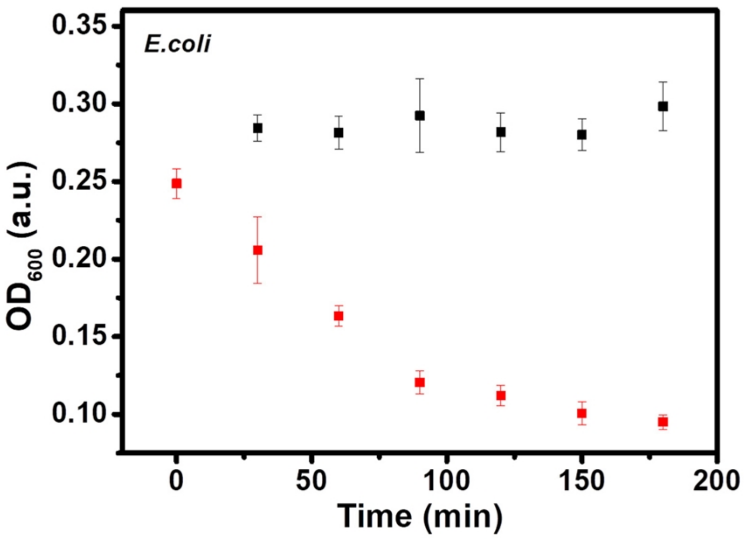

Figure S9. The bacterial density of the *E.coli* incubated with (red dots) and without (black dots) GBFC-powered patch for different time. The bacterial density was examined via recording the optical density at 600 nm.

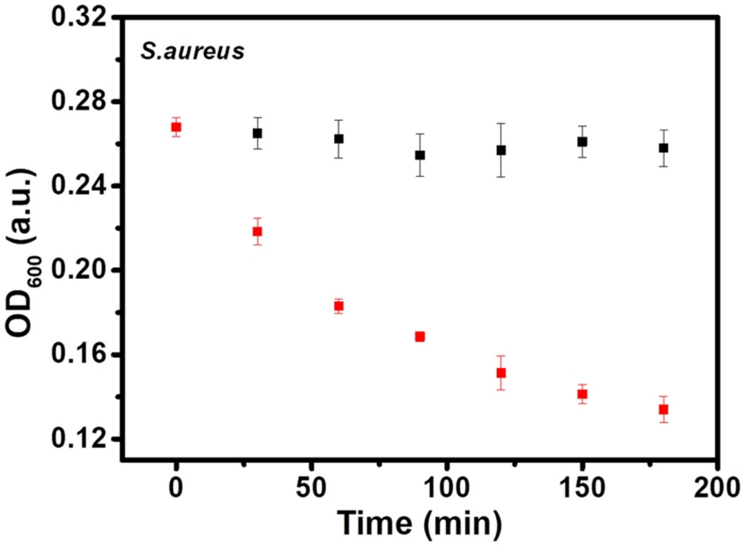

Figure S10. The bacterial density of the *S. aureus* incubated with (red dots) and without (black dots) GBFC-powered patch for different time. The bacterial density was examined via recording the optical density at 600 nm.

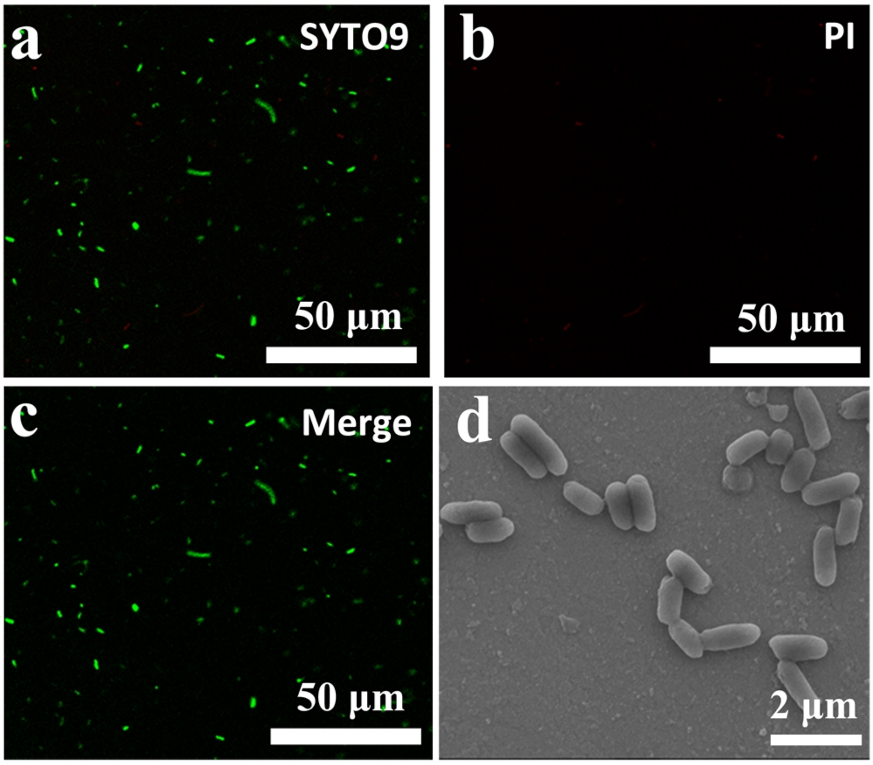

Figure S11. The CLSM images (a-c) and the SEM image (d) of the residual *E.coli* collected from the bacteria culture medium incubated with inactive enzyme modified patch for 3 h (green fluorescence: live bacteria stained with SYTO9, red fluorescence: dead bacteria stained with PI).

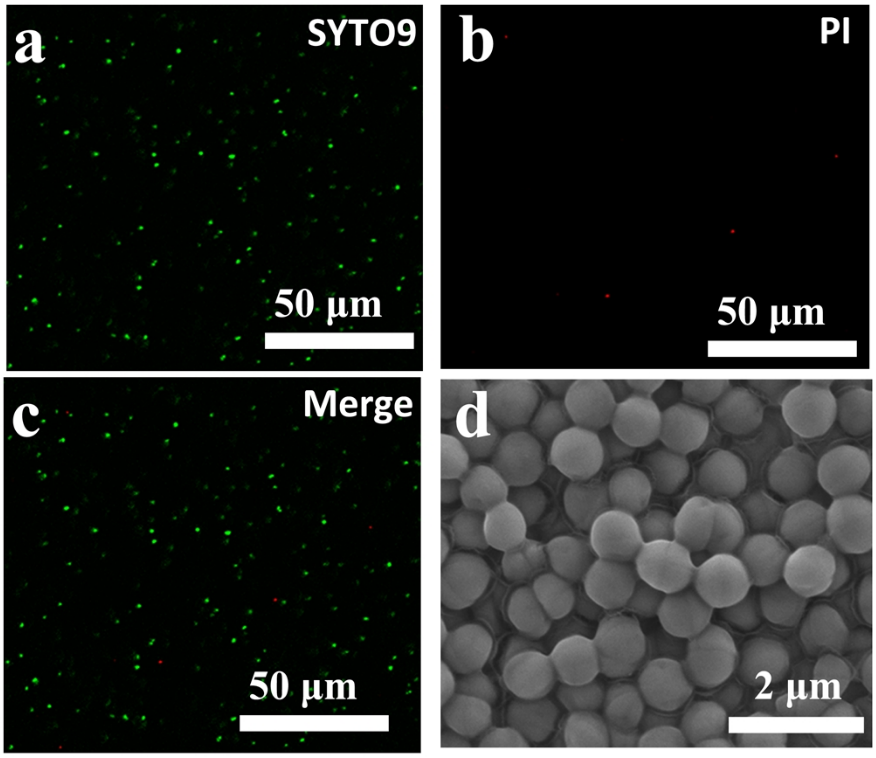

Figure S12. CLSM images (a-c) and the SEM image (d) of the residual *S. aureus* collected from the bacteria culture medium incubated with inactive enzyme modified patch for 3 h (green fluorescence: live bacteria stained with SYTO9, red fluorescence: dead bacteria stained with PI).

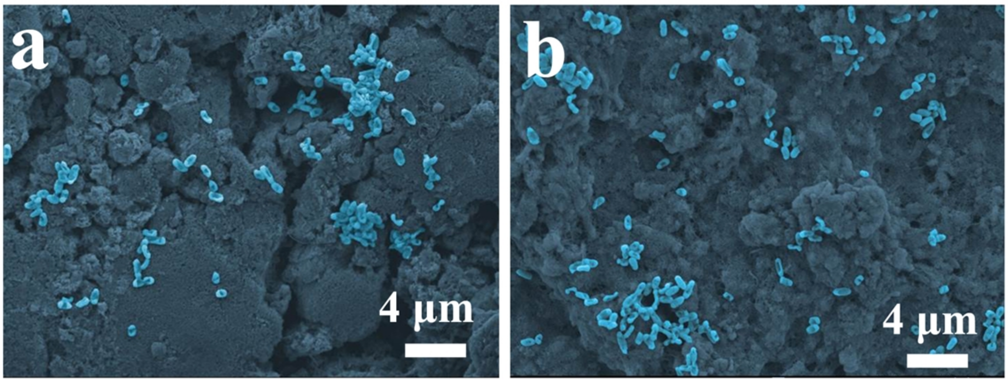

Figure S13. SEM images of the open-circuit patch's electrodes incubated with *E.coli* for 3 h. (a) anode; (b) cathode.

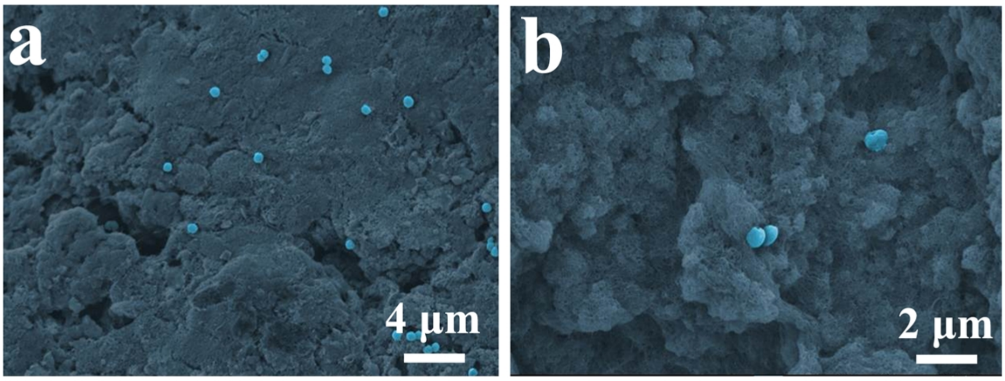

Figure S14. SEM images of the open-circuit patch's electrodes incubated with *S. aureus* for 3 h. (a) anode; (b) cathode.

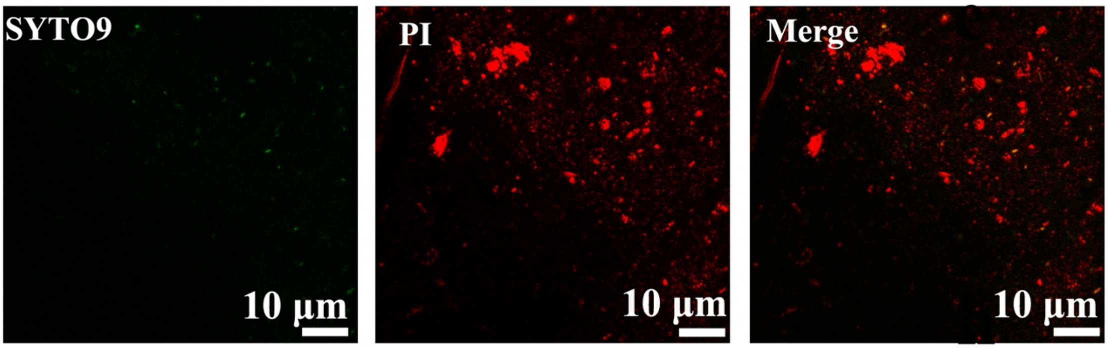

Figure S15. The CLSM images of the patch's cathode incubated with *E.coli* for 3 h (green fluorescence: live bacteria stained with SYTO9, red fluorescence: dead bacteria stained with PI).

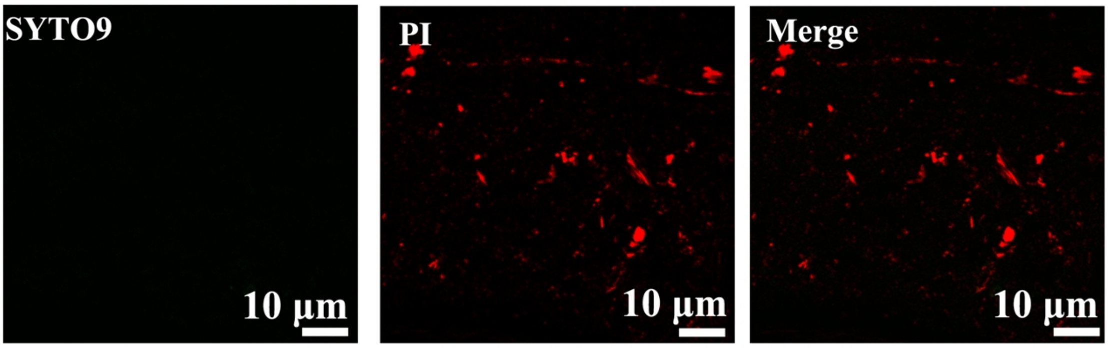

Figure S16. The CLSM images of the patch's cathode incubated with *S. aureus* for 3 h. (green fluorescence: live bacteria stained with SYTO9, red fluorescence: dead bacteria stained with PI).

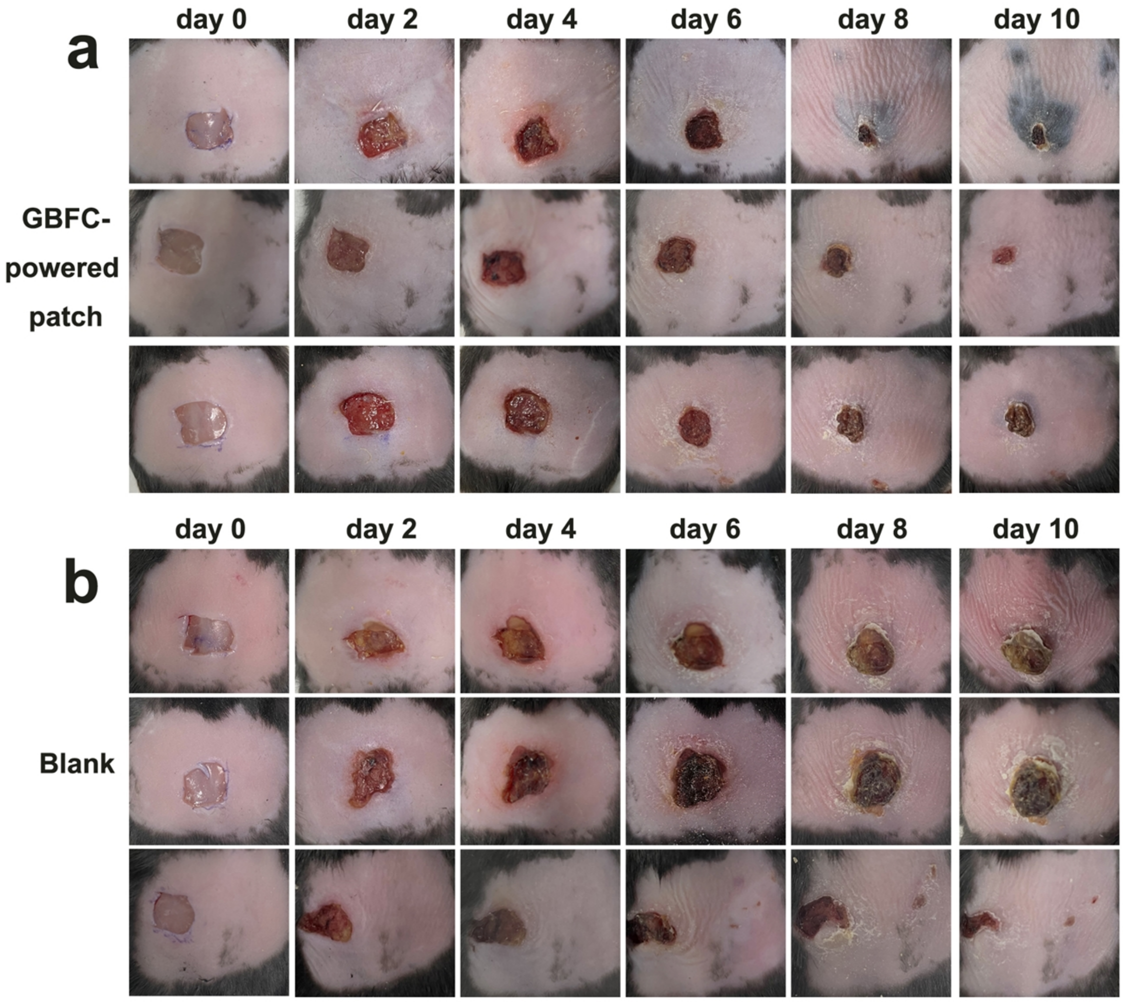

Figure S17. Photographs of the *S. aureus*-infected diabetic wounds treated by GBFC-powered patch (a) and PBS (b) for different time.

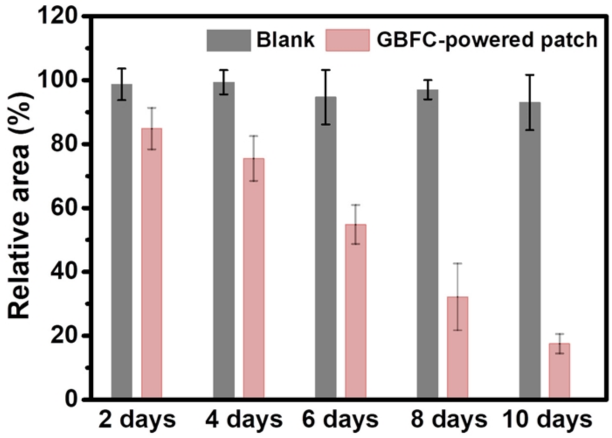

Figure S18. The closure rate of the wounds on the diabetic mice treated by PBS and

the GBFC-powered patch.

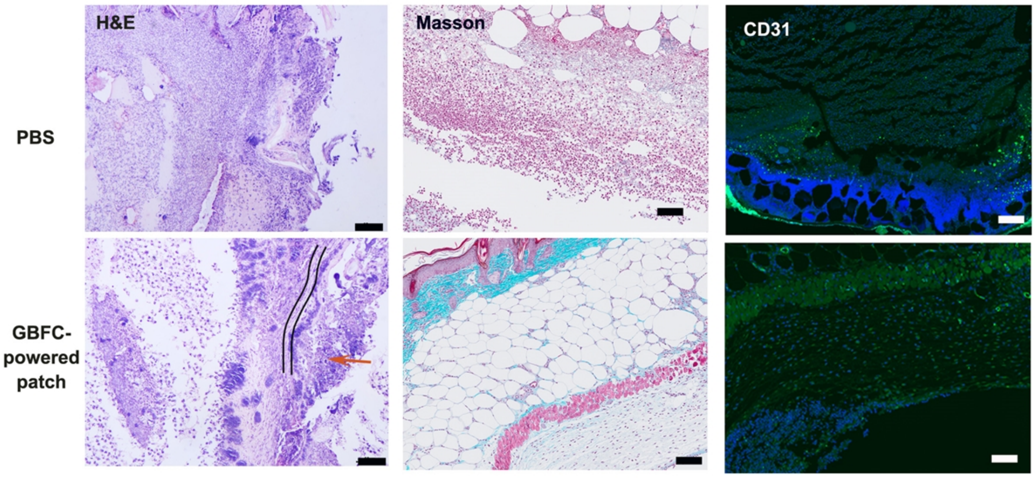

Figure S19. Histologic analysis of the bacteria-infected wounds treated by PBS and GBFC-powered patch for 3 days. H&E, Masson and CD31 staining were used to investigate the inflammation response, collagen deposition and neovascularization in wound area, respectively. Scale bars: 50 µm (H&E), 100 µm (Masson), 100 µm (CD31).
